# Clinical Characteristics of Emergency Visits Related to Recreational Psychedelic Use

**DOI:** 10.1007/s10597-026-01620-x

**Published:** 2026-04-01

**Authors:** Kush V. Bhatt, Joseph Friedman, Lindsay Benster, Rekha Narasimhan, Kevin Yang, Jyoti Mishra, Cory R. Weissman, Dhakshin Ramanathan

**Affiliations:** University of California, San Diego, San Diego, United States

**Keywords:** Psychedelic, hallucinogen, emergency department, substance use

## Abstract

Recreational psychedelic use is increasing, yet data on adverse events remains limited. This study characterized emergency department (ED) visits associated with recreational psychedelic use at UC San Diego Medical Center. We conducted a retrospective chart review of ED encounters (2010–2023). Cases were identified using ICD-10 hallucinogen-related codes and confirmed through manual review. Multivariable logistic regression was employed to identify factors associated with psychiatric hospitalization. We identified 232 cases, primarily related to LSD (35.0%), MDMA (30.2%), and psilocybin (24.0%). The cohort was predominantly young, white, and male. Common psychiatric symptoms included agitation (25.9%) and anxiety (24.6%). Common nonpsychiatric symptoms included nausea/vomiting (9.5%) and diaphoresis (5.2%). 11.2% of cases required psychiatric hospitalization. Factors associated with psychiatric hospitalization included concurrent cannabis use (OR = 10.9, 95% CI 3.37–39.64), history of bipolar disorder (OR = 12.67, 95% CI 2.35–70.43), and history of a primary psychotic disorder (OR = 17.10, 95% CI 2.01–187.49). Psychedelic-associated emergency visits present with various clinical characteristics. While most recreational psychedelic presentations are effectively managed in the emergency department, specific factors predict severe outcomes. Concurrent cannabis use and pre-existing psychotic or bipolar disorders are associated with increased odds of psychiatric hospitalization, underscoring the importance of targeted risk assessment in patients with these vulnerabilities.

**Supplementary Information:**

The online version contains supplementary material available at https://doi.org/10.1007/s10597-026-01620-x.

## Introduction

Psychedelics are powerful consciousness-altering substances with a complex history. While studies of the therapeutic use of psychedelics date back to the 1950s, research advances were stunted when psychedelics were classified as Schedule 1 substances in the United States. Recently, there has been a resurgence of interest from scientists and clinicians (Carhart-Harris & Goodwin, 2017; Goodwin et al., 2022; Griffiths et al., 2008; Raison et al., 2023) due to promising findings on their therapeutic effects in neuropsychiatric disorders, and the recognized need for novel and effective treatments (Grover et al., 2023). Parallel to this is a rise in public interest and recreational consumption of these substances (Gearin & Devenot, 2021), driven in part by policy shifts including FDA breakthrough therapy designation for psilocybin and MDMA-assisted therapy and decriminalization initiatives in multiple U.S. jurisdictions. The NIH-funded Monitoring The Future (MTF) study, which has tracked psychedelic use since 1975, has identified a rapid rise in recreational psychedelic use that appears particularly pronounced in recent year. (Keyes & Patrick, 2023; Patrick et al., n.d.).

With the rapid rise of psychedelic use, it is important to better understand the potential associated risks (McNamee et al., 2023; Simonsson et al., 2023a, 2023b). Recent clinical trials have shown that psychedelics are generally safe (Yao et al., 2024) with minimal adverse effects (Hinkle et al., 2024). However, psychedelic clinical trials exclude individuals with known or presumed risk factors, and use rigorous medical surveillance and psychological guidance. These conditions create favorable safety conditions that do not clearly reflect recreational use (Romeo et al., 2024). Psychiatric risks associated with recreational psychedelic use include suicidal ideation, hallucinogen persisting perception disorder (HPPD), and psychosis (Schlag et al., 2022). Medical risks have largely focused on concerns that 5HT-2B receptor agonism may increase the risk of valvular heart disease (Tagen et al., 2023). However, most evidence to date has been gathered from case reports and observational studies.

Thus, despite the potential clinical benefits, psychedelic use in nonclinical settings raises significant concern for safety. One way of understanding acute risk is to survey emergency department (ED) visits associated with psychedelic use. A recent study found a significant rise in ED visits related to hallucinogen use (Garel et al., 2024). However, this study lacked specificity for psychedelics, as the term hallucinogen encompasses both psychedelics and nonpsychedelic hallucinogens such as phencyclidine, ketamine, salvia, and dextromethorphan. Currently, the clinical characteristics of individuals who present to the ED after using psychedelics remain poorly defined. Our study examined the clinical characteristics and factors associated with psychiatric hospitalization among individuals presenting to the emergency department following psychedelic use. We focused primarily on classical psychedelics — substances that act as agonists at the 5-HT2A receptor — and additionally included MDMA, given its serotonergic mechanism, its FDA Breakthrough Therapy designation for PTSD, and its frequent co-classification as a psychedelic in both research and recreational contexts. We hypothesized that pre-existing psychiatric history and concurrent substance use would be associated with increased odds of psychiatric hospitalization following psychedelic-related ED presentation.

## Methods

### Data Source and Case Identification

We used data available in the electronic health records (EPIC, Verona, WI, USA) from University of California, San Diego Medical Center, a large academic hospital system in Southern California. We aggregated potential psychedelic-associated adverse events by identifying ED visits in which a diagnosis of hallucinogen-related disorder (ICD-10 code F16*) was made during that specific encounter between January 2010 and October 2023. ICD-10 F16* codes were selected as they represent the most specific available coding category for psychedelic-associated presentations, acknowledging that psychedelic-related visits may also be coded under other categories (e.g., F19* for polysubstance use), potentially resulting in missed cases. EPIC slicer dicer was used to identify patients and subsequently search charts. An initial review was conducted by one of the authors (KB) by searching the following keywords within the narrative component of emergency provider documentation: “psychedelic” “MDMA” “Molly” “Ecstacy” “Psilocybin” “Mushrooms” “LSD” “Acid” “Peyote” “Mescaline” “Ayahuasca” “DMT.” Authors KB, LB, and RN then conducted a review of ED visits considered relevant from the keyword search by utilizing the “ED Provider Note” which is a standardized health record note with all available clinical information for that specific patient visit. Data that was considered relevant was extracted to a Ready Electronic Data Capture (REDCap) survey. Cases in which psilocybin, LSD, ayahuasca, DMT, and MDMA were associated, were identified as psychedelic-associated cases. The criteria to be considered a psychedelic-associated visit required the following 1) psychedelic drug use was reported by the patient or collateral source AND 2) the treating emergency room physician explicitly assessed the cause of the presentation to be at least partially associated with psychedelic use, as described in the narrative assessment. Cases meeting only one criterion were excluded, and any disagreements in case identification were ultimately adjudicated by author KB. Concurrent substance use (cannabis, alcohol, methamphetamine, cocaine, opioid) were identified using both self/collateral report and urine toxicology screening. Each included case corresponded to a unique medical record number, ensuring that only one visit per patient was analyzed. If there was a possibility of multiple psychedelic-associated ED visits for one individual, only the first instance was included. De-identified data was then converted to a data sheet for statistical analysis.

### Ethical Considerations

This retrospective analysis was deemed IRB Exempt by the University of California, San Diego IRB Committee (IRB#809537).

### Data Extraction and Variable Definitions

Variables of interest were extracted from provider documentation in the ED provider note, including demographics, psychiatric symptoms, nonpsychiatric symptoms, past psychiatric history, concurrent substance use, and vital signs. Vital sign abnormalities were categorized as mild, moderate, or severe using standardized clinical thresholds (see Supplementary Methods for detailed definitions).

Missing data for demographic variables were minimal (≤ 1.3% across all variables). For clinical variables including psychiatric symptoms, nonpsychiatric symptoms, and past psychiatric history, absence of documentation in the ED provider note was coded as absence of the finding, consistent with standard practice in retrospective chart review studies.

### Statistical Analysis Plan

All analyses were performed using R version 4.4.1. Descriptive statistics were calculated for clinical characteristics. Multivariable logistic regression was employed to assess the relationship between probability of psychiatric hospitalization and explanatory factors chosen a priori*,* including concurrent substance use, past psychiatric history, and sociodemographic factors. As this was a retrospective exploratory study, a formal power calculation was not performed.

## Results

We identified 674 cases with an ICD-10 code for hallucinogen-related disorders (F16*), of which 232 were confirmed to be related to recreational psychedelic use. 296 were excluded as the hallucinogen-related diagnosis reflected a historical use disorder not relevant to the current ED presentation. Of the remaining 378 cases, 146 were excluded as they were related to non-psychedelic hallucinogens (phencyclidine, n = 99; ketamine, n = 29; other hallucinogen, n = 18) This yielded a final sample of 232 confirmed psychedelic-associated ED visits. LSD was the most frequently identified psychedelic substance (35.0%), followed by MDMA (30.2%) and psilocybin (24.0%). Polysubstance use involving multiple psychedelics was less common, with LSD and MDMA co-use accounting for 6.9% of cases.

### Demographic Characteristics

Overall, patients were predominantly male (69.4%) and white (53.4%). The mean age was 26.3 ± 7.7 years. Among psilocybin-associated cases, the average age was 27.2 ± 7.01 years, with 76.4% of cases involving male patients. For LSD-related cases, the average age was slightly younger at 25.9 ± 9.15 years, with 75.3% being male patients. MDMA-related cases had an average of 25.9 ± 6.49 years, with 60.0% male (Table 1).

**Table 1:** Demographic information

	**LSD**	**MDMA**	**Psilocybin**	**MDMA + LSD**	**Other**	**Overall**
	**(N = 81)**	**(N = 70)**	**(N = 55)**	**(N = 16)**	**(N = 10)**	**(N = 232)**
***Demographics and Hospitalization Status***						
**Age**						
Mean (SD)	25.9 (9.15)	25.9 (6.49)	27.2 (7.01)	23.9 (4.59)	31.1 (9.45)	26.3 (7.71)
Median [Min, Max]	23.0 [14.0, 63.0]	24.0 [16.0, 52.0]	26.0 [16.0, 48.0]	23.0 [19.0, 36.0]	32.0 [18.0, 47.0]	24.0 [14.0, 63.0]
Missing	1 (1.2%)	0 (0%)	0 (0%)	1 (6.3%)	1 (10.0%)	3 (1.3%)
**Gender**						
Male	61 (75.3%)	42 (60.0%)	42 (76.4%)	10 (62.5%)	6 (60.0%)	161 (69.4%)
Female	20 (24.7%)	27 (38.6%)	13 (23.6%)	6 (37.5%)	4 (40.0%)	70 (30.2%)
Missing	0 (0%)	1 (1.4%)	0 (0%)	0 (0%)	0 (0%)	1 (0.4%)
**Race**						
White	47 (58.0%)	30 (42.9%)	30 (54.5%)	10 (62.5%)	7 (70.0%)	124 (53.4%)
Black	1 (1.2%)	2 (2.9%)	4 (7.3%)	2 (12.5%)	0 (0%)	9 (3.9%)
Asian	9 (11.1%)	17 (24.3%)	4 (7.3%)	1 (6.3%)	0 (0%)	31 (13.4%)
Unknown/Undocumented	23 (28.4%)	20 (28.6%)	17 (30.9%)	3 (18.8%)	3 (30.0%)	66 (28.4%)
Missing	1 (1.2%)	1 (1.4%)	0 (0%)	0 (0%)	0 (0%)	2 (0.9%)

### Psychiatric and Nonpsychiatric Symptoms and Vital Sign Abnormalities

**Fig. 1:**
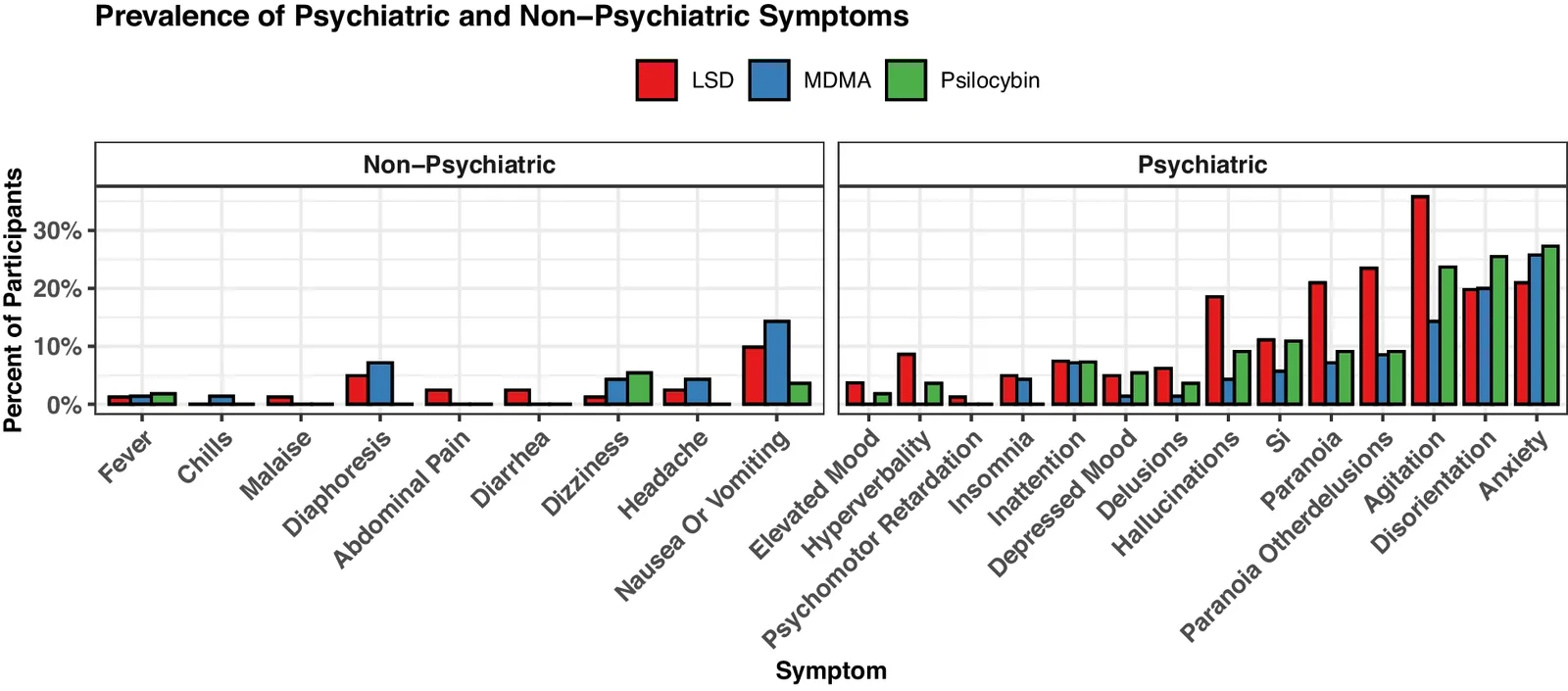
Prevalence of psychiatric and non-psychiatric symptoms

Psychiatric symptoms were more common than nonpsychiatric symptoms. Agitation (25.9%) and anxiety (24.6%) were the most frequently reported symptoms across the total cohort. Symptom profiles varied slightly by substance: agitation was the predominant symptom in LSD cases (35.8%), whereas anxiety was the leading symptom in MDMA (25.7%) and psilocybin (27.3%) presentations. Nonpsychiatric symptoms were less frequent, with nausea/vomiting (9.5%) and diaphoresis (5.2%) being the most common (Fig. 1).

Hemodynamic changes were notable; mild diastolic hypotension (24.6%) and systolic hypertension (20.3%) were frequently observed. Tachycardia was prevalent, with 12.1% of patients presenting with severe tachycardia, a finding most frequently observed with MDMA use (See Supplementary Table for more details).

### Past Psychiatric History and Concurrent Substance Use

**Fig. 2:**
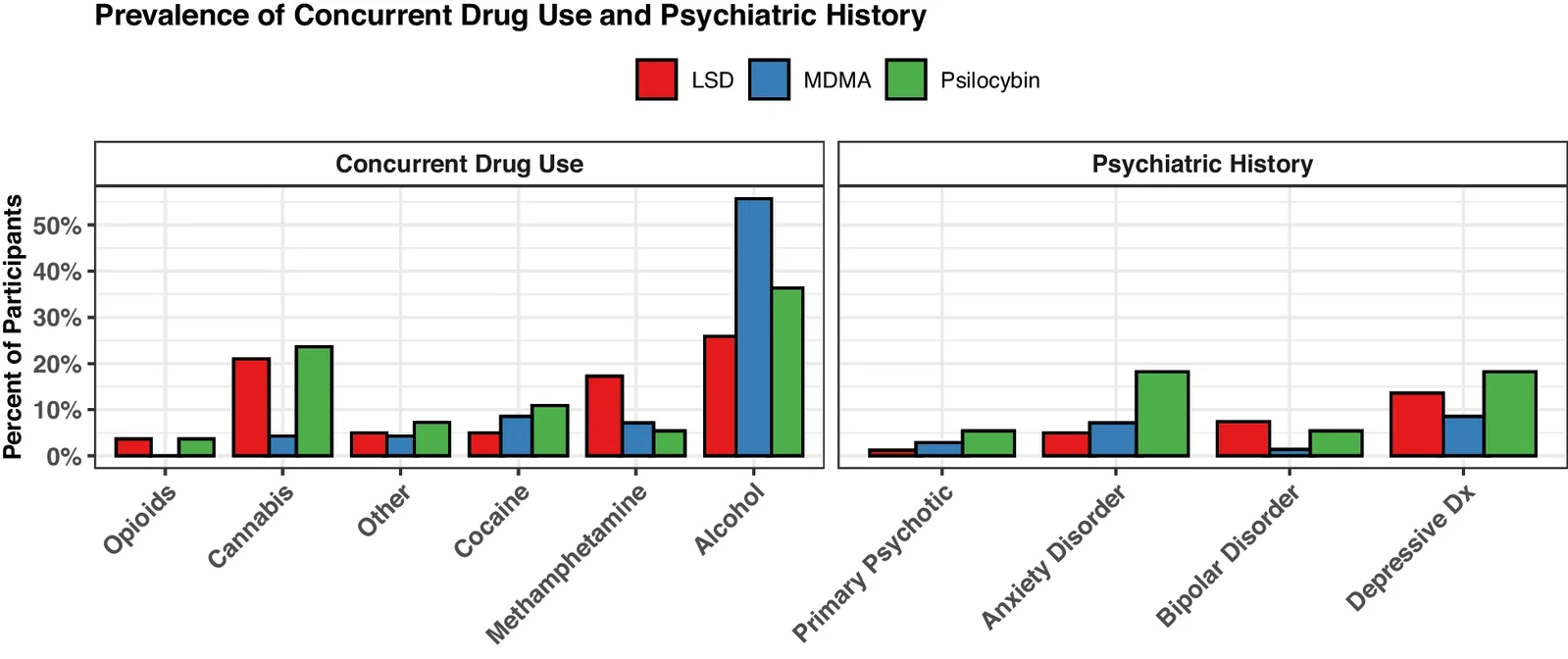
Prevalence of concurrent drug use and psychiatric history

A significant portion of the cohort had a documented psychiatric history. Unipolar depression was the most common diagnosis (13.8%), followed by anxiety disorders (8.2%) and bipolar spectrum disorders (5.2%). Concurrent substance use was highly prevalent; 37.9% of cases involved alcohol co-ingestion, and 18.5% involved cannabis. Concurrent alcohol use was particularly high in MDMA-related visits (55.7%) (Fig. 2).

### Factors Associated with Psychiatric Hospitalization

**Fig. 3:**
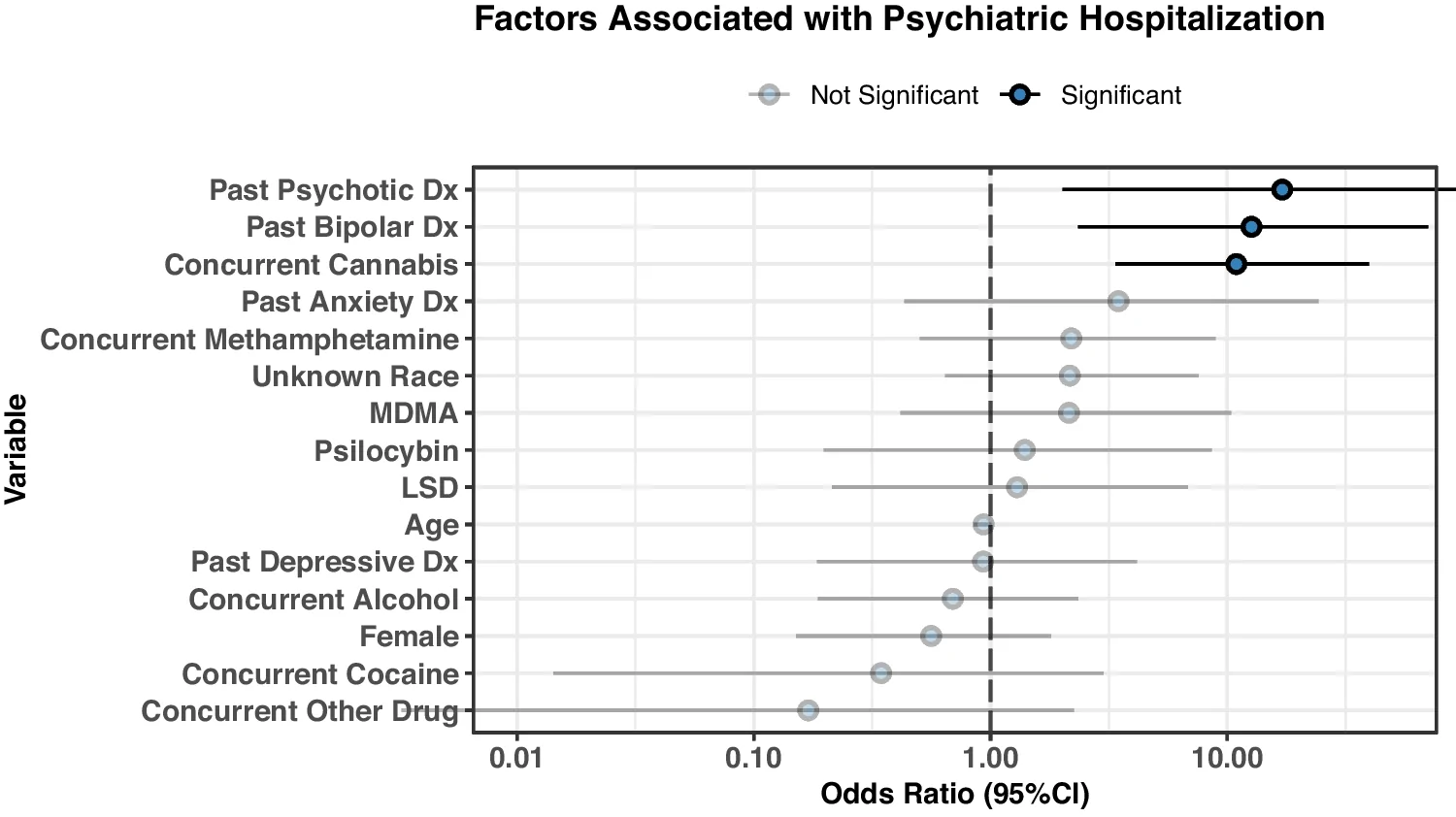
Factors associated with psychiatric hospitalization

While the majority of patients were discharged from the ED, 11.2% required psychiatric hospitalization. In multivariable models, concurrent cannabis use (OR = 10.9, 95% CI 3.37–39.64), history of bipolar disorder (OR = 12.67, 95% CI 2.35–70.43), and history of a primary psychotic disorder (OR = 17.10, 95% CI 2.01–187.49) were significantly associated with increased odds of psychiatric hospitalization (Fig. 3). Other variables included in the multivariable model, including age, sex, race, substance type (LSD, psilocybin, MDMA), concurrent alcohol, cocaine, and methamphetamine use, and history of depressive and anxiety disorders, were not significantly associated with psychiatric hospitalization. Confidence intervals were wide across predictor variables; see Limitations for further discussion.

## Discussion

As interest in psychedelic drugs continues to grow, understanding potential adverse effects related to psychedelic use is critical. Recent clinical trials suggest that psychedelics, namely LSD, MDMA, and psilocybin, are generally safe treatments; however, given the increased recreational use of these substances, understanding risks of use in uncontrolled settings is critically needed. Our study explored the clinical characteristics of emergency visits associated with recreational psychedelic use. We identified 232 cases of emergency visits that were at least partially attributed to the use of these substances. Our findings highlight key clinical characteristics associated with psychedelic-related emergency visits.

### Psychedelic Use Pattern

The majority of identified cases were related to LSD, followed by MDMA and psilocybin, a finding consistent with previous research suggesting that LSD, MDMA, and psilocybin are the most used psychedelics recreationally (Lake & Lucas, 2024b). We also identified co-use of psychedelics in some cases. Most commonly we identified co-use of LSD and MDMA, a common recreational combination colloquially termed “candy flipping.” The prevalence of LSD/MDMA co-use in our ED cohort (6.9%) was at the lower end of the 8–52% range reported among recreational psychedelic users in other studies (Zeifman et al., 2023), suggesting that such a combination does not necessarily disproportionately drive emergency presentations relative to its known prevalence in recreational use. Interestingly, research suggests that this combination may actually enhance positive effects and mitigates challenging psychedelic experiences (Zeifman et al., 2023). However, it may lead to more potent vital sign abnormalities including hypertension and tachycardia (Straumann et al., 2023). Overall, the risks of this combination are not well known, but some research suggests it may increase risk of chronic perceptual disturbances (Lake & Lucas, 2024a; Müller et al., 2022).

### Sociodemographic

The majority of cases were young adults, predominantly male, and of white race. These demographic patterns align with previous data, which suggests that young white men are more likely to utilize psychedelic drugs (Krebs & Johansen, 2013; Sexton et al., 2019). This population generally shows higher rates of risky drug use. Moreover, adolescence and early adulthood represents a critical period for both substance use initiation and development of psychiatric disorders, making this group particularly vulnerable to adverse outcomes from psychedelic use (Kessler et al., 2007).

### Symptom Profile in Recreational vs. Clinical Context

The symptom profiles of identified cases are consistent with prior findings (Kopra et al., 2022) that psychedelics carry both physiological and psychological risk, particularly in vulnerable populations (Rossi et al., 2022). Frequently reported psychiatric symptoms included anxiety, hallucinations, paranoid delusions, disorientation, and agitation. Frequently reported nonpsychiatric symptoms included nausea, vomiting, dizziness, headache, diaphoresis, and hemodynamic changes. A key insight from these findings is that there is overlap between symptoms observed in individuals in clinical trials and in those who received emergency care for recreational use (Yerubandi et al., 2024; Yildirim et al., 2024). In a controlled clinical environment, these effects can likely be managed through psychological guidance and close medical monitoring. Moreover some of these symptoms, namely anxiety and hallucinations are often framed as part of the therapeutic process (Aqil & Roseman, 2023; Viljoen & Betzler, 2024). This underscores that the favorable safety profile observed in clinical trials may be contingent upon the structured setting and exclusion of high-risk co-morbidities and substance use, conditions that are likely absent in the recreational sphere.

### Psychiatric Hospitalization

While the majority of patients were discharged, some required psychiatric hospitalization. Our multivariable regression analysis identified three factors associated with increased odds of psychiatric admission: a history of bipolar disorder, a history of primary psychotic disorder, and concurrent cannabis use.

The association between pre-existing psychiatric disorders and hospitalization highlights the vulnerability of this population. Psychedelic drugs have been noted to induce decompensation in those with previous psychiatric history, particularly those with bipolar disorder and psychotic disorders (Gard et al., 2021; Sabé et al., 2024). For this reason, psychedelic clinical trials have excluded individuals with bipolar disorder and psychotic disorders, except for some investigation of psilocybin-assisted therapy for bipolar 2 depression (Aaronson et al., 2024). Notably, current evidence suggests that psychedelics are unlikely to directly induce bipolar disorder or psychotic disorders, as research indicates low incidence of primary psychotic disorders triggered by psychedelics, even when transient psychotic symptoms occur (Honk et al., 2024; Simonsson et al., 2023a, 2023b). Individuals with a family history of psychotic disorders or bipolar disorder appear to be at increased risk, however.

It is notable that while depression was the most common diagnosis in our cohort, it was not a significant predictor of hospitalization, whereas bipolar and psychotic disorders were. This finding likely reflects both the high prevalence of depression in the general population, and the lower risk for severe decompensation with psychedelics. Interestingly, a recent study identified an association between depression and recreational LSD use, raising the question of whether individuals with depression are more likely to use LSD or whether LSD use may contribute to depressive symptoms (Walsh et al., 2024). Clarifying this directionality is important for distinguishing the effects of recreational versus clinically supervised psychedelic use on psychiatric outcomes.

### The Role of Concurrent Substance Use

A significant proportion of individuals who presented with psychedelic-associated adverse events had simultaneously consumed other psychoactive substances, most frequently alcohol, cannabis, and methamphetamine. Co-ingested substances may have exacerbated the severity of symptoms related to psychedelics and contributed to the need for emergency care. We found that cannabis use was associated with increased odds of psychiatric hospitalization. The specific association between cannabis and psychiatric hospitalization, rather than other co-used substances, may reflect the synergistic effects of cannabis on serotonergic and endocannabinoid systems, amplifying the perceptual and psychological effects of psychedelics and increasing the likelihood of challenging experiences (Kuc et al., 2022). Whether this association is dose-dependent, timing-dependent, or moderated by individual vulnerability factors remains unclear. Interestingly, recent research indicates that cannabis co-use is common among psychedelic users in general, not just those experiencing adverse effects (Lake & Lucas, 2024a). These findings in combinations with ours suggest that the relationship between cannabis and psychedelic use requires further inquiry.

### Limitations and Future Directions

There are several important limitations to our study. First, this study relied on emergency department electronic health records, which may not always accurately or completely capture relevant clinical information. Cases were identified using an ICD-10 diagnostic code and keywords, which may have missed relevant emergency cases. Thus, the data from this study should be interpreted with caution. Second, psychedelic use was determined based on self or collateral report, without toxicology confirmation. Moreover, the doses and purity of substances was unknown. Third, this study was conducted at a single academic hospital, and only captured individuals who sought emergency care, limiting generalizability of our findings. Fourth, given the high prevalence of polysubstance use in our cohort, attribution of clinical presentations specifically to psychedelics is uncertain, as alcohol, cannabis, and methamphetamine can independently produce similar symptoms and commonly precipitate emergency department visits, which limits our ability to isolate psychedelic-specific effects (Lewer et al., 2020; Nguyen et al., 2024; Pickens et al., 2022; White et al., 2018). Finally, confidence intervals for all predictor variables were wide, again highlighting the need for cautious interpretation of these findings. They likely reflect the small sample size and heterogeneity of the clinical cases, including variability in the frequency of different variables and lack of statistical power due to the exploratory nature of the study.

Future research should aim to replicate these findings in larger, multi-site samples to address the limitations to generalizability of the current study. Case identification by employing keyword-based searches of provider notes or extended toxicology screening would likely serve as a more sensitive capture strategy than ICD-10 coding.

## Conclusion

This study explored the clinical characteristics of psychedelic-associated emergency visits. Our findings add to a growing body of research exploring the potential risks of recreational psychedelic use. With the rapid rise in psychedelic use both recreationally and in medical settings, there is a need for better understanding of risks. Our study identified clinical characteristics, including psychiatric and nonpsychiatric symptoms, vital sign abnormalities, past psychiatric history, and concurrent substance use were present with varying frequency for individuals who sought emergency care for psychedelic use. Although most patients were discharged from the emergency department, some required hospitalization. We found that history of psychotic disorder or bipolar disorder, and concurrent cannabis use were predictors of psychiatric hospitalization. These findings, while preliminary, have implications for medical providers who should maintain heightened awareness of psychiatric history and concurrent substance use when evaluating psychedelic-related presentations. Specifically, individuals with histories of bipolar disorder or psychotic disorders would benefit from psychoeducation regarding the elevated risks of recreational psychedelic use, along with the risks of concurrent substance use. As psychedelics continue to garner interest both as therapeutic agents and recreational substances, a nuanced understanding of their risk profile — particularly in vulnerable populations and uncontrolled settings — is essential to ensuring that their potential benefits are realized safely and equitably.

## Supplementary Information

Below is the link to the electronic supplementary material.

Supplementary file1 (DOCX 14 KB)

Supplementary file2 (XLSX 13 KB)

## Data Availability

All data supporting the findings of this study are available from the corresponding author upon reasonable request.
