# Supplementary material for: Clinical Characteristics of Emergency Visits Related to Recreational Psychedelic Use
Source: Community Ment Health J. 2026 Apr 1;62(7):1252–60. doi: 10.1007/s10597-026-01620-x (PMC13585822; doi:10.1007/s10597-026-01620-x)
Supplement: Supplementary file 1 [file 10597_2026_1620_MOESM1_ESM.docx]

*Supplementary Methods:*

Definitions of variables used in descriptive analysis:

Hypotension was defined as: mild (85-89 mmHg systolic and/or 60-70 mmHg diastolic), moderate (80-84mmHg systolic and/or 50-59 mmHg diastolic), and severe (less than 80 mmHg systolic or less than 50 mmHg diastolic ). Hypertension was defined as: mild (141-159mmHg systolic and/or 91-99 mmHg), moderate (160-179 mmHg systolic and/or 100-109 mmHg), and severe (greater than 180 mmHg systolic and/or greater 110 mmHg diastolic). Tachycardia was defined as: mild (101-115 beats/min), moderate (116-130 beats/min), and severe (greater than 130 beats/min). Fever was defined as temperature greater than 37.6 degrees Fahrenheit.

The absence of documentation of a specific symptom was interpreted as the absence of that symptom. Past psychiatric history was determined based on documented prior diagnoses in the ED provider note. Concurrent substance use was defined as the documented use of other substances during the same episode leading to the emergency visit, as reported by the patient, collateral source, or toxicology screening.

Psychiatric symptoms were categorized according to standardized DSM-5 terminology, and medical symptoms were categorized by organ systems.
